# 1-(Pyridin-2-yl)-2-[2-(trifluoro­meth­yl)benz­yl]-3-[2-(trifluoro­meth­yl)phen­yl]propan-1-one

**DOI:** 10.1107/S1600536812003698

**Published:** 2012-02-04

**Authors:** Muhammad Naveed Umar, Mohammad Shoaib, Seik Weng Ng

**Affiliations:** a School of Chemistry, University of Malakand, Khyber Pakhtunkhwa, Pakistan; b Department of Chemistry, University of Malaya, 50603 Kuala Lumpur, Malaysia, and Chemistry Department, Faculty of Science, King Abdulaziz University, PO Box 80203 Jeddah, Saudi Arabia

## Abstract

The title compound, C_23_H_17_F_6_NO, crystallizes with two mol­ecules in the asymmetric unit. The mol­ecules assume an approximate propellar shape, with the three aromatic rings being bent with respect to the plane formed by the C atoms that are connected to the methine C atom [dihedral angles: pyridyl 67.49 (3)°, phenyl 56.82 (4)° and phenyl 77.21 (6)° in one mol­ecule, and corresponding angles of 71.60 (6), 53.68 (4) and 77.53 (6)° in the second mol­ecule].

## Related literature

For 2-benzyl-3-phenyl-1-(pyridin-2-yl)propan-1-one, see: Naveed Umar *et al.* (2012 ▶).
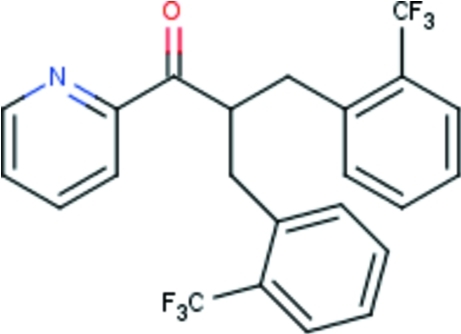


## Experimental

### 

#### Crystal data

C_23_H_17_F_6_NO
*M* *_r_* = 437.38
Triclinic, 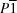

*a* = 8.0661 (3) Å
*b* = 12.4900 (7) Å
*c* = 20.8047 (11) Å
α = 106.101 (5)°
β = 98.366 (3)°
γ = 91.609 (3)°
*V* = 1987.17 (17) Å^3^
*Z* = 4
Cu *K*α radiation
μ = 1.12 mm^−1^
*T* = 100 K
0.40 × 0.30 × 0.20 mm

#### Data collection

Agilent SuperNova Dual diffractometer with Atlas detector
Absorption correction: multi-scan (*CrysAlis PRO*; Agilent, 2011 ▶) *T*_min_ = 0.664, *T*_max_ = 0.808
24362 measured reflections
8257 independent reflections
7494 reflections with *I* > 2σ(*I*)
*R*_int_ = 0.036

#### Refinement

*R*[*F* ^2^ > 2σ(*F* ^2^)] = 0.050
*wR*(*F* ^2^) = 0.151
*S* = 1.05
8257 reflections
560 parameters
H-atom parameters constrained
Δρ_max_ = 0.37 e Å^−3^
Δρ_min_ = −0.32 e Å^−3^

### 

Data collection: *CrysAlis PRO* (Agilent, 2011 ▶); cell refinement: *CrysAlis PRO*; data reduction: *CrysAlis PRO*; program(s) used to solve structure: *SHELXS97* (Sheldrick, 2008 ▶); program(s) used to refine structure: *SHELXL97* (Sheldrick, 2008 ▶); molecular graphics: *X-SEED* (Barbour, 2001 ▶); software used to prepare material for publication: *publCIF* (Westrip, 2010 ▶).

## Supplementary Material

Crystal structure: contains datablock(s) global, I. DOI: 10.1107/S1600536812003698/bt5804sup1.cif

Structure factors: contains datablock(s) I. DOI: 10.1107/S1600536812003698/bt5804Isup2.hkl

Supplementary material file. DOI: 10.1107/S1600536812003698/bt5804Isup3.cml

Additional supplementary materials:  crystallographic information; 3D view; checkCIF report

## Figures and Tables

**Fig. 1.**
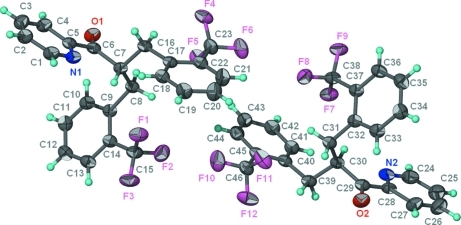
Anisotropic displacement ellipsoid plot (Barbour, 2001) of the two independent molecules of C23H17F6NO at the 70% probability level; hydrogen atoms are drawn as spheres of arbitrary radius.

## supplementary crystallographic information

### Comment

2-Benzyl-3-phenyl-1-(pyridin-2-yl)propan-1-one, in the optically active form, was synthesized for use in fast aldol condensations. The racemic molecule assumes an approximate propellar shape, with the three aromatic rings being nearly perpendicularly aligned at with respect to the plane formed by the C atoms that are connected to the methine C atom (Naveed Umar *et al.*, 2012). The title trifluomethyl-substituted analog (Scheme I) crystallizes with two molecules in the asymmetric unit (Fig. 1). The C_23_H_17_F_6_NO molecule assumes an approximate propellar shape, with the three aromatic rings being bent with respect to the plane formed by the C atoms that are connected to the methine C atom [dihedral angles: pyridyl 67.49 (3), phenyl 56.82 (4), phenyl 77.21 (6) ° in one molecule and corresponding angles of 71.60 (6), 53.68 (4) and 77.53 (6) ° in the second molecule].

### Experimental

In a 250 ml flask was added sodium borohydride (4 equiv, 1.2 mg, 48 mmol) in anhydrous toluene (40 ml) followed by the addition of 18-crown-6 (0.1 equiv, 0.32 mg, 1.2 mmol), and acetyl pyridine (1 equiv, 1.35 ml, 12 mmol). Bromomethyl-2-(trifluoromethyl)benzene (2.5 equiv, 30 mmol) was added. The reaction mixture was stirred at 323 K for 5 h under an inert atmosphere. The reaction was monitored by TLC and GC. The reaction was quenched by adding saturated ammonium chloride. The organic compound was extracted with ethyl acetate. The organic layer was dried over sodium sulfate and the solvent removed to give a yellow oil. This was submitted to flash chromatography and eluted with 5% ethyl acetate/hexane to give the desired ketone product (75% yield).

### Refinement

H-atoms were placed in calculated positions [C–H 0.95 to 0.99 Å, *U*_iso_(H) 1.2*U*_eq_(C)] and were included in the refinement in the riding model approximation.

### Crystal data

**Table:**

C_23_H_17_F_6_NO	*Z* = 4
*M**_r_* = 437.38	*F*(000) = 896
Triclinic, *P*1	*D*_x_ = 1.462 Mg m^−^^3^
Hall symbol: -P 1	Cu *K*α radiation, λ = 1.54184 Å
*a* = 8.0661 (3) Å	Cell parameters from 11397 reflections
*b* = 12.4900 (7) Å	θ = 3.7–76.2°
*c* = 20.8047 (11) Å	µ = 1.12 mm^−^^1^
α = 106.101 (5)°	*T* = 100 K
β = 98.366 (3)°	Block, colourless
γ = 91.609 (3)°	0.40 × 0.30 × 0.20 mm
*V* = 1987.17 (17) Å^3^	

### Data collection

**Table:**

Agilent SuperNova Dual diffractometer with Atlas detector	8257 independent reflections
Radiation source: SuperNova (Cu) X-ray Source	7494 reflections with *I* > 2σ(*I*)
Mirror monochromator	*R*_int_ = 0.036
Detector resolution: 10.4041 pixels mm^-1^	θ_max_ = 76.4°, θ_min_ = 3.7°
ω scans	*h* = −8→10
Absorption correction: multi-scan (*CrysAlis PRO*; Agilent, 2011)	*k* = −14→15
*T*_min_ = 0.664, *T*_max_ = 0.808	*l* = −26→25
24362 measured reflections	

### Refinement

**Table:**

Refinement on *F*^2^	Secondary atom site location: difference Fourier map
Least-squares matrix: full	Hydrogen site location: inferred from neighbouring sites
*R*[*F*^2^ > 2σ(*F*^2^)] = 0.050	H-atom parameters constrained
*wR*(*F*^2^) = 0.151	*w* = 1/[σ^2^(*F*_o_^2^) + (0.0963*P*)^2^ + 0.5056*P*] where *P* = (*F*_o_^2^ + 2*F*_c_^2^)/3
*S* = 1.05	(Δ/σ)_max_ = 0.001
8257 reflections	Δρ_max_ = 0.37 e Å^−^^3^
560 parameters	Δρ_min_ = −0.32 e Å^−^^3^
0 restraints	Extinction correction: *SHELXL97* (Sheldrick, 2008), Fc^*^=kFc[1+0.001xFc^2^λ^3^/sin(2θ)]^-1/4^
Primary atom site location: structure-invariant direct methods	Extinction coefficient: 0.0067 (6)

### Fractional atomic coordinates and isotropic or equivalent isotropic displacement parameters (Å^2^)

**Table:**

	*x*	*y*	*z*	*U*_iso_*/*U*_eq_	
F1	0.29915 (13)	0.49876 (8)	0.24587 (5)	0.0392 (2)	
F2	0.48970 (14)	0.48674 (9)	0.18214 (6)	0.0440 (3)	
F3	0.23550 (14)	0.51272 (9)	0.14497 (5)	0.0460 (3)	
F4	0.84263 (12)	0.45553 (9)	0.46684 (5)	0.0417 (3)	
F5	0.84693 (11)	0.48650 (9)	0.37019 (5)	0.0372 (2)	
F6	0.90031 (12)	0.32509 (9)	0.38276 (6)	0.0445 (3)	
F7	1.08148 (12)	0.03173 (8)	0.24782 (5)	0.0364 (2)	
F8	0.88657 (13)	0.04809 (9)	0.30988 (5)	0.0403 (2)	
F9	1.13471 (13)	0.01151 (9)	0.34784 (5)	0.0394 (2)	
F10	0.43671 (12)	0.22454 (10)	0.16205 (7)	0.0492 (3)	
F11	0.49491 (11)	0.05632 (9)	0.15518 (6)	0.0421 (3)	
F12	0.47286 (12)	0.11462 (11)	0.06717 (6)	0.0470 (3)	
O1	0.59731 (13)	0.82502 (10)	0.45935 (6)	0.0320 (3)	
O2	0.73087 (14)	−0.25571 (10)	0.02599 (6)	0.0354 (3)	
N1	0.17930 (15)	0.71875 (11)	0.44649 (6)	0.0279 (3)	
N2	1.16198 (16)	−0.17349 (12)	0.04471 (6)	0.0290 (3)	
C1	0.03704 (19)	0.74806 (14)	0.47062 (8)	0.0318 (3)	
H1	−0.0575	0.6952	0.4562	0.038*	
C2	0.01926 (19)	0.85093 (15)	0.51538 (8)	0.0339 (3)	
H2	−0.0849	0.8681	0.5306	0.041*	
C3	0.1571 (2)	0.92794 (15)	0.53734 (9)	0.0346 (3)	
H3	0.1492	0.9992	0.5680	0.042*	
C4	0.30691 (19)	0.89923 (13)	0.51385 (8)	0.0308 (3)	
H4	0.4040	0.9499	0.5286	0.037*	
C5	0.31185 (17)	0.79472 (12)	0.46828 (7)	0.0253 (3)	
C6	0.47017 (17)	0.76226 (12)	0.43984 (7)	0.0250 (3)	
C7	0.46772 (17)	0.64771 (12)	0.38948 (7)	0.0246 (3)	
H7	0.3493	0.6227	0.3671	0.030*	
C8	0.57695 (17)	0.64823 (12)	0.33463 (7)	0.0255 (3)	
H8A	0.6911	0.6813	0.3568	0.031*	
H8B	0.5871	0.5703	0.3079	0.031*	
C9	0.50383 (17)	0.71396 (13)	0.28721 (7)	0.0252 (3)	
C10	0.54330 (18)	0.82873 (13)	0.30524 (8)	0.0286 (3)	
H10	0.6176	0.8631	0.3462	0.034*	
C11	0.47736 (19)	0.89424 (14)	0.26521 (9)	0.0316 (3)	
H11	0.5064	0.9724	0.2790	0.038*	
C12	0.36912 (19)	0.84620 (14)	0.20509 (8)	0.0313 (3)	
H12	0.3228	0.8911	0.1778	0.038*	
C13	0.32924 (18)	0.73178 (14)	0.18524 (8)	0.0304 (3)	
H13	0.2565	0.6979	0.1438	0.037*	
C14	0.39532 (17)	0.66639 (13)	0.22569 (8)	0.0264 (3)	
C15	0.3547 (2)	0.54255 (14)	0.19996 (8)	0.0325 (3)	
C16	0.52929 (18)	0.56981 (13)	0.43279 (7)	0.0265 (3)	
H16A	0.6518	0.5856	0.4481	0.032*	
H16B	0.4740	0.5869	0.4736	0.032*	
C17	0.49401 (17)	0.44675 (13)	0.39549 (7)	0.0249 (3)	
C18	0.32545 (18)	0.40471 (13)	0.37760 (8)	0.0283 (3)	
H18	0.2395	0.4540	0.3884	0.034*	
C19	0.28157 (19)	0.29295 (14)	0.34449 (9)	0.0346 (4)	
H19	0.1666	0.2663	0.3329	0.042*	
C20	0.4052 (2)	0.22001 (14)	0.32829 (10)	0.0401 (4)	
H20	0.3750	0.1435	0.3051	0.048*	
C21	0.5730 (2)	0.25874 (14)	0.34593 (10)	0.0363 (4)	
H21	0.6580	0.2087	0.3352	0.044*	
C22	0.61688 (17)	0.37129 (13)	0.37939 (8)	0.0276 (3)	
C23	0.80011 (18)	0.40896 (14)	0.39969 (9)	0.0318 (3)	
C24	1.3028 (2)	−0.21222 (15)	0.02317 (8)	0.0345 (4)	
H24	1.4014	−0.1629	0.0363	0.041*	
C25	1.3139 (2)	−0.31965 (17)	−0.01703 (9)	0.0403 (4)	
H25	1.4176	−0.3436	−0.0305	0.048*	
C26	1.1699 (2)	−0.39148 (17)	−0.03713 (10)	0.0446 (4)	
H26	1.1728	−0.4657	−0.0649	0.054*	
C27	1.0217 (2)	−0.35315 (15)	−0.01601 (9)	0.0365 (4)	
H27	0.9210	−0.4005	−0.0292	0.044*	
C28	1.02335 (18)	−0.24427 (13)	0.02484 (7)	0.0269 (3)	
C29	0.86514 (18)	−0.20152 (13)	0.04974 (7)	0.0269 (3)	
C30	0.87595 (17)	−0.09058 (12)	0.10449 (7)	0.0256 (3)	
H30	0.9965	−0.0671	0.1241	0.031*	
C31	0.77939 (17)	−0.10214 (13)	0.16118 (7)	0.0265 (3)	
H31A	0.7726	−0.0268	0.1921	0.032*	
H31B	0.6632	−0.1328	0.1406	0.032*	
C32	0.85667 (17)	−0.17599 (13)	0.20236 (8)	0.0262 (3)	
C33	0.81346 (18)	−0.29045 (14)	0.18028 (8)	0.0305 (3)	
H33	0.7386	−0.3206	0.1390	0.037*	
C34	0.8769 (2)	−0.36182 (14)	0.21702 (9)	0.0332 (3)	
H34	0.8460	−0.4397	0.2005	0.040*	
C35	0.98530 (19)	−0.31946 (15)	0.27782 (9)	0.0339 (3)	
H35	1.0279	−0.3679	0.3033	0.041*	
C36	1.03093 (18)	−0.20567 (14)	0.30106 (8)	0.0313 (3)	
H36	1.1051	−0.1760	0.3426	0.038*	
C37	0.96848 (17)	−0.13511 (13)	0.26371 (8)	0.0274 (3)	
C38	1.01779 (19)	−0.01234 (14)	0.29190 (8)	0.0315 (3)	
C39	0.80054 (18)	−0.00242 (13)	0.07124 (7)	0.0278 (3)	
H39	0.8488	−0.0069	0.0295	0.033*	
H39B	0.6776	−0.0193	0.0581	0.033*	
C40	0.83571 (17)	0.11498 (13)	0.11867 (7)	0.0260 (3)	
C41	1.00266 (18)	0.15960 (13)	0.13416 (8)	0.0290 (3)	
H41	1.0858	0.1170	0.1130	0.035*	
C42	1.05035 (19)	0.26379 (14)	0.17930 (9)	0.0325 (3)	
H42	1.1646	0.2919	0.1885	0.039*	
C43	0.9310 (2)	0.32732 (13)	0.21115 (9)	0.0328 (3)	
H43	0.9634	0.3981	0.2431	0.039*	
C44	0.76404 (19)	0.28638 (14)	0.19582 (8)	0.0316 (3)	
H44	0.6816	0.3299	0.2169	0.038*	
C45	0.71607 (18)	0.18158 (13)	0.14962 (8)	0.0278 (3)	
C46	0.53164 (18)	0.14473 (14)	0.13360 (8)	0.0309 (3)	

### Atomic displacement parameters (Å^2^)

**Table:**

	*U*^11^	*U*^22^	*U*^33^	*U*^12^	*U*^13^	*U*^23^
F1	0.0467 (6)	0.0329 (5)	0.0340 (5)	−0.0169 (4)	−0.0041 (4)	0.0103 (4)
F2	0.0484 (6)	0.0323 (5)	0.0448 (6)	0.0011 (4)	0.0048 (5)	0.0016 (4)
F3	0.0527 (6)	0.0406 (6)	0.0332 (5)	−0.0150 (5)	−0.0175 (4)	0.0060 (4)
F4	0.0275 (4)	0.0525 (6)	0.0403 (5)	−0.0034 (4)	−0.0119 (4)	0.0145 (5)
F5	0.0232 (4)	0.0398 (5)	0.0507 (6)	−0.0050 (4)	0.0034 (4)	0.0181 (4)
F6	0.0221 (4)	0.0431 (6)	0.0694 (7)	0.0059 (4)	0.0041 (4)	0.0192 (5)
F7	0.0399 (5)	0.0351 (5)	0.0304 (5)	−0.0129 (4)	−0.0013 (4)	0.0084 (4)
F8	0.0415 (5)	0.0368 (5)	0.0371 (5)	0.0046 (4)	0.0030 (4)	0.0029 (4)
F9	0.0400 (5)	0.0423 (6)	0.0281 (5)	−0.0076 (4)	−0.0082 (4)	0.0053 (4)
F10	0.0240 (5)	0.0437 (6)	0.0746 (8)	0.0056 (4)	0.0046 (5)	0.0097 (5)
F11	0.0243 (4)	0.0465 (6)	0.0625 (7)	−0.0046 (4)	0.0026 (4)	0.0298 (5)
F12	0.0253 (5)	0.0724 (8)	0.0400 (6)	−0.0088 (5)	−0.0104 (4)	0.0198 (5)
O1	0.0235 (5)	0.0329 (6)	0.0332 (6)	−0.0092 (4)	0.0001 (4)	0.0022 (4)
O2	0.0236 (5)	0.0388 (6)	0.0355 (6)	−0.0085 (4)	−0.0027 (4)	0.0019 (5)
N1	0.0219 (6)	0.0316 (7)	0.0269 (6)	−0.0053 (5)	−0.0005 (5)	0.0060 (5)
N2	0.0238 (6)	0.0356 (7)	0.0261 (6)	−0.0044 (5)	−0.0007 (5)	0.0092 (5)
C1	0.0221 (7)	0.0373 (8)	0.0315 (7)	−0.0059 (6)	−0.0008 (6)	0.0055 (6)
C2	0.0243 (7)	0.0411 (9)	0.0333 (8)	0.0013 (6)	0.0037 (6)	0.0064 (7)
C3	0.0315 (8)	0.0340 (8)	0.0336 (8)	−0.0009 (6)	0.0037 (6)	0.0031 (6)
C4	0.0264 (7)	0.0313 (8)	0.0300 (7)	−0.0055 (6)	0.0005 (6)	0.0038 (6)
C5	0.0218 (6)	0.0279 (7)	0.0241 (7)	−0.0033 (5)	−0.0021 (5)	0.0071 (5)
C6	0.0218 (6)	0.0274 (7)	0.0244 (7)	−0.0039 (5)	−0.0018 (5)	0.0081 (5)
C7	0.0191 (6)	0.0271 (7)	0.0244 (7)	−0.0040 (5)	−0.0016 (5)	0.0051 (5)
C8	0.0196 (6)	0.0288 (7)	0.0260 (7)	−0.0024 (5)	−0.0010 (5)	0.0072 (5)
C9	0.0183 (6)	0.0299 (7)	0.0267 (7)	−0.0033 (5)	0.0026 (5)	0.0079 (6)
C10	0.0231 (6)	0.0317 (8)	0.0285 (7)	−0.0052 (5)	0.0021 (5)	0.0061 (6)
C11	0.0287 (7)	0.0296 (8)	0.0375 (8)	−0.0022 (6)	0.0069 (6)	0.0109 (6)
C12	0.0260 (7)	0.0365 (8)	0.0358 (8)	0.0009 (6)	0.0051 (6)	0.0177 (7)
C13	0.0230 (7)	0.0382 (8)	0.0299 (7)	−0.0027 (6)	−0.0005 (5)	0.0122 (6)
C14	0.0213 (6)	0.0293 (7)	0.0273 (7)	−0.0034 (5)	0.0002 (5)	0.0084 (6)
C15	0.0331 (8)	0.0331 (8)	0.0278 (7)	−0.0067 (6)	−0.0045 (6)	0.0084 (6)
C16	0.0231 (6)	0.0290 (7)	0.0240 (7)	−0.0040 (5)	−0.0028 (5)	0.0062 (6)
C17	0.0210 (6)	0.0293 (7)	0.0238 (6)	−0.0035 (5)	−0.0013 (5)	0.0094 (5)
C18	0.0205 (6)	0.0326 (8)	0.0315 (7)	−0.0017 (5)	0.0009 (5)	0.0106 (6)
C19	0.0215 (7)	0.0351 (8)	0.0444 (9)	−0.0068 (6)	−0.0013 (6)	0.0109 (7)
C20	0.0299 (8)	0.0279 (8)	0.0565 (11)	−0.0049 (6)	0.0030 (7)	0.0049 (7)
C21	0.0256 (7)	0.0316 (8)	0.0501 (10)	0.0006 (6)	0.0053 (7)	0.0093 (7)
C22	0.0206 (7)	0.0317 (8)	0.0302 (7)	−0.0028 (5)	−0.0011 (5)	0.0115 (6)
C23	0.0217 (7)	0.0322 (8)	0.0414 (9)	0.0002 (6)	0.0000 (6)	0.0132 (7)
C24	0.0257 (7)	0.0469 (10)	0.0296 (8)	−0.0035 (6)	0.0025 (6)	0.0105 (7)
C25	0.0311 (8)	0.0519 (11)	0.0363 (9)	0.0052 (7)	0.0079 (7)	0.0084 (8)
C26	0.0426 (9)	0.0431 (10)	0.0401 (9)	0.0032 (8)	0.0071 (7)	−0.0016 (8)
C27	0.0324 (8)	0.0361 (9)	0.0326 (8)	−0.0068 (6)	0.0000 (6)	−0.0002 (7)
C28	0.0243 (7)	0.0331 (8)	0.0209 (6)	−0.0035 (6)	−0.0018 (5)	0.0072 (6)
C29	0.0224 (6)	0.0308 (7)	0.0251 (7)	−0.0047 (5)	−0.0027 (5)	0.0082 (6)
C30	0.0196 (6)	0.0286 (7)	0.0251 (7)	−0.0039 (5)	−0.0022 (5)	0.0055 (6)
C31	0.0203 (6)	0.0298 (7)	0.0274 (7)	−0.0010 (5)	−0.0007 (5)	0.0074 (6)
C32	0.0193 (6)	0.0320 (8)	0.0268 (7)	−0.0016 (5)	0.0028 (5)	0.0082 (6)
C33	0.0243 (7)	0.0334 (8)	0.0319 (8)	−0.0047 (6)	0.0009 (6)	0.0085 (6)
C34	0.0307 (7)	0.0302 (8)	0.0395 (9)	−0.0017 (6)	0.0055 (6)	0.0115 (7)
C35	0.0276 (7)	0.0390 (9)	0.0395 (9)	0.0025 (6)	0.0053 (6)	0.0182 (7)
C36	0.0230 (7)	0.0414 (9)	0.0298 (7)	−0.0014 (6)	0.0006 (5)	0.0130 (6)
C37	0.0213 (6)	0.0328 (8)	0.0272 (7)	−0.0023 (5)	0.0022 (5)	0.0086 (6)
C38	0.0291 (7)	0.0367 (8)	0.0252 (7)	−0.0040 (6)	−0.0010 (6)	0.0063 (6)
C39	0.0236 (6)	0.0318 (8)	0.0253 (7)	−0.0050 (5)	−0.0029 (5)	0.0078 (6)
C40	0.0217 (6)	0.0297 (7)	0.0263 (7)	−0.0031 (5)	−0.0017 (5)	0.0107 (6)
C41	0.0218 (7)	0.0312 (8)	0.0331 (8)	−0.0029 (5)	0.0009 (5)	0.0100 (6)
C42	0.0229 (7)	0.0316 (8)	0.0402 (8)	−0.0069 (6)	−0.0031 (6)	0.0108 (7)
C43	0.0299 (7)	0.0278 (7)	0.0361 (8)	−0.0031 (6)	−0.0032 (6)	0.0063 (6)
C44	0.0273 (7)	0.0324 (8)	0.0341 (8)	0.0014 (6)	0.0006 (6)	0.0103 (6)
C45	0.0213 (7)	0.0320 (8)	0.0296 (7)	−0.0027 (5)	−0.0028 (5)	0.0120 (6)
C46	0.0224 (7)	0.0344 (8)	0.0363 (8)	0.0005 (6)	0.0000 (6)	0.0131 (6)

### Geometric parameters (Å, º)

**Table:**

F1—C15	1.3463 (19)	C18—C19	1.384 (2)
F2—C15	1.351 (2)	C18—H18	0.9500
F3—C15	1.3401 (18)	C19—C20	1.384 (2)
F4—C23	1.344 (2)	C19—H19	0.9500
F5—C23	1.3542 (18)	C20—C21	1.386 (2)
F6—C23	1.3417 (19)	C20—H20	0.9500
F7—C38	1.3449 (18)	C21—C22	1.394 (2)
F8—C38	1.3540 (19)	C21—H21	0.9500
F9—C38	1.3436 (18)	C22—C23	1.5011 (19)
F10—C46	1.333 (2)	C24—C25	1.384 (3)
F11—C46	1.3433 (18)	C24—H24	0.9500
F12—C46	1.3369 (19)	C25—C26	1.386 (3)
O1—C6	1.2199 (17)	C25—H25	0.9500
O2—C29	1.2218 (18)	C26—C27	1.386 (3)
N1—C1	1.339 (2)	C26—H26	0.9500
N1—C5	1.3463 (18)	C27—C28	1.388 (2)
N2—C24	1.336 (2)	C27—H27	0.9500
N2—C28	1.3454 (19)	C28—C29	1.503 (2)
C1—C2	1.387 (2)	C29—C30	1.521 (2)
C1—H1	0.9500	C30—C31	1.541 (2)
C2—C3	1.385 (2)	C30—C39	1.549 (2)
C2—H2	0.9500	C30—H30	1.0000
C3—C4	1.388 (2)	C31—C32	1.513 (2)
C3—H3	0.9500	C31—H31A	0.9900
C4—C5	1.390 (2)	C31—H31B	0.9900
C4—H4	0.9500	C32—C33	1.392 (2)
C5—C6	1.504 (2)	C32—C37	1.407 (2)
C6—C7	1.519 (2)	C33—C34	1.389 (2)
C7—C8	1.5411 (19)	C33—H33	0.9500
C7—C16	1.545 (2)	C34—C35	1.388 (2)
C7—H7	1.0000	C34—H34	0.9500
C8—C9	1.517 (2)	C35—C36	1.388 (2)
C8—H8A	0.9900	C35—H35	0.9500
C8—H8B	0.9900	C36—C37	1.387 (2)
C9—C10	1.392 (2)	C36—H36	0.9500
C9—C14	1.4073 (19)	C37—C38	1.501 (2)
C10—C11	1.386 (2)	C39—C40	1.516 (2)
C10—H10	0.9500	C39—H39	0.9900
C11—C12	1.387 (2)	C39—H39B	0.9900
C11—H11	0.9500	C40—C41	1.4000 (19)
C12—C13	1.387 (2)	C40—C45	1.402 (2)
C12—H12	0.9500	C41—C42	1.383 (2)
C13—C14	1.391 (2)	C41—H41	0.9500
C13—H13	0.9500	C42—C43	1.390 (2)
C14—C15	1.499 (2)	C42—H42	0.9500
C16—C17	1.515 (2)	C43—C44	1.386 (2)
C16—H16A	0.9900	C43—H43	0.9500
C16—H16B	0.9900	C44—C45	1.397 (2)
C17—C22	1.399 (2)	C44—H44	0.9500
C17—C18	1.4025 (19)	C45—C46	1.5060 (19)
			
C1—N1—C5	116.80 (14)	F5—C23—C22	112.71 (12)
C24—N2—C28	116.94 (14)	N2—C24—C25	124.07 (15)
N1—C1—C2	123.98 (14)	N2—C24—H24	118.0
N1—C1—H1	118.0	C25—C24—H24	118.0
C2—C1—H1	118.0	C24—C25—C26	118.27 (16)
C3—C2—C1	118.39 (15)	C24—C25—H25	120.9
C3—C2—H2	120.8	C26—C25—H25	120.9
C1—C2—H2	120.8	C27—C26—C25	118.87 (17)
C2—C3—C4	118.87 (15)	C27—C26—H26	120.6
C2—C3—H3	120.6	C25—C26—H26	120.6
C4—C3—H3	120.6	C26—C27—C28	118.67 (15)
C3—C4—C5	118.56 (14)	C26—C27—H27	120.7
C3—C4—H4	120.7	C28—C27—H27	120.7
C5—C4—H4	120.7	N2—C28—C27	123.18 (14)
N1—C5—C4	123.37 (14)	N2—C28—C29	117.11 (13)
N1—C5—C6	116.55 (13)	C27—C28—C29	119.70 (13)
C4—C5—C6	120.07 (13)	O2—C29—C28	119.96 (14)
O1—C6—C5	120.19 (14)	O2—C29—C30	121.16 (14)
O1—C6—C7	121.90 (13)	C28—C29—C30	118.87 (12)
C5—C6—C7	117.83 (12)	C29—C30—C31	110.29 (12)
C6—C7—C8	112.20 (11)	C29—C30—C39	108.14 (11)
C6—C7—C16	105.09 (11)	C31—C30—C39	110.65 (12)
C8—C7—C16	112.61 (12)	C29—C30—H30	109.2
C6—C7—H7	108.9	C31—C30—H30	109.2
C8—C7—H7	108.9	C39—C30—H30	109.2
C16—C7—H7	108.9	C32—C31—C30	114.50 (12)
C9—C8—C7	112.15 (12)	C32—C31—H31A	108.6
C9—C8—H8A	109.2	C30—C31—H31A	108.6
C7—C8—H8A	109.2	C32—C31—H31B	108.6
C9—C8—H8B	109.2	C30—C31—H31B	108.6
C7—C8—H8B	109.2	H31A—C31—H31B	107.6
H8A—C8—H8B	107.9	C33—C32—C37	117.13 (14)
C10—C9—C14	117.09 (14)	C33—C32—C31	119.32 (13)
C10—C9—C8	118.88 (13)	C37—C32—C31	123.53 (13)
C14—C9—C8	124.03 (13)	C32—C33—C34	121.80 (14)
C11—C10—C9	121.89 (14)	C32—C33—H33	119.1
C11—C10—H10	119.1	C34—C33—H33	119.1
C9—C10—H10	119.1	C35—C34—C33	120.07 (15)
C10—C11—C12	120.28 (15)	C35—C34—H34	120.0
C10—C11—H11	119.9	C33—C34—H34	120.0
C12—C11—H11	119.9	C34—C35—C36	119.42 (15)
C13—C12—C11	119.21 (14)	C34—C35—H35	120.3
C13—C12—H12	120.4	C36—C35—H35	120.3
C11—C12—H12	120.4	C37—C36—C35	120.16 (14)
C12—C13—C14	120.31 (14)	C37—C36—H36	119.9
C12—C13—H13	119.8	C35—C36—H36	119.9
C14—C13—H13	119.8	C36—C37—C32	121.41 (14)
C13—C14—C9	121.22 (14)	C36—C37—C38	118.14 (14)
C13—C14—C15	117.89 (13)	C32—C37—C38	120.40 (14)
C9—C14—C15	120.82 (13)	F7—C38—F9	106.09 (12)
F3—C15—F1	105.87 (12)	F7—C38—F8	105.94 (13)
F3—C15—F2	106.05 (13)	F9—C38—F8	105.81 (13)
F1—C15—F2	105.95 (13)	F7—C38—C37	113.10 (13)
F3—C15—C14	112.94 (13)	F9—C38—C37	112.73 (13)
F1—C15—C14	113.23 (13)	F8—C38—C37	112.56 (13)
F2—C15—C14	112.21 (13)	C40—C39—C30	111.80 (11)
C17—C16—C7	113.58 (11)	C40—C39—H39	109.3
C17—C16—H16A	108.9	C30—C39—H39	109.3
C7—C16—H16A	108.8	C40—C39—H39B	109.3
C17—C16—H16B	108.8	C30—C39—H39B	109.3
C7—C16—H16B	108.8	H39—C39—H39B	107.9
H16A—C16—H16B	107.7	C41—C40—C45	117.27 (14)
C22—C17—C18	117.57 (14)	C41—C40—C39	117.12 (13)
C22—C17—C16	124.96 (12)	C45—C40—C39	125.59 (12)
C18—C17—C16	117.45 (13)	C42—C41—C40	122.05 (15)
C19—C18—C17	121.43 (14)	C42—C41—H41	119.0
C19—C18—H18	119.3	C40—C41—H41	119.0
C17—C18—H18	119.3	C41—C42—C43	119.94 (14)
C18—C19—C20	120.03 (14)	C41—C42—H42	120.0
C18—C19—H19	120.0	C43—C42—H42	120.0
C20—C19—H19	120.0	C44—C43—C42	119.35 (15)
C19—C20—C21	119.93 (16)	C44—C43—H43	120.3
C19—C20—H20	120.0	C42—C43—H43	120.3
C21—C20—H20	120.0	C43—C44—C45	120.52 (15)
C20—C21—C22	119.94 (15)	C43—C44—H44	119.7
C20—C21—H21	120.0	C45—C44—H44	119.7
C22—C21—H21	120.0	C44—C45—C40	120.82 (13)
C21—C22—C17	121.10 (13)	C44—C45—C46	117.39 (14)
C21—C22—C23	118.20 (14)	C40—C45—C46	121.77 (14)
C17—C22—C23	120.67 (14)	F10—C46—F12	106.10 (13)
F6—C23—F4	106.48 (12)	F10—C46—F11	105.90 (13)
F6—C23—F5	105.85 (13)	F12—C46—F11	105.76 (13)
F4—C23—F5	106.02 (13)	F10—C46—C45	112.65 (14)
F6—C23—C22	112.80 (13)	F12—C46—C45	112.92 (13)
F4—C23—C22	112.44 (13)	F11—C46—C45	112.90 (12)
			
C5—N1—C1—C2	0.7 (2)	C28—N2—C24—C25	−0.8 (2)
N1—C1—C2—C3	−0.8 (3)	N2—C24—C25—C26	0.9 (3)
C1—C2—C3—C4	−0.1 (2)	C24—C25—C26—C27	−0.3 (3)
C2—C3—C4—C5	1.0 (2)	C25—C26—C27—C28	−0.3 (3)
C1—N1—C5—C4	0.3 (2)	C24—N2—C28—C27	0.1 (2)
C1—N1—C5—C6	−178.72 (13)	C24—N2—C28—C29	179.20 (13)
C3—C4—C5—N1	−1.2 (2)	C26—C27—C28—N2	0.4 (3)
C3—C4—C5—C6	177.83 (14)	C26—C27—C28—C29	−178.62 (16)
N1—C5—C6—O1	−177.00 (13)	N2—C28—C29—O2	169.67 (14)
C4—C5—C6—O1	3.9 (2)	C27—C28—C29—O2	−11.2 (2)
N1—C5—C6—C7	−0.06 (19)	N2—C28—C29—C30	−10.31 (19)
C4—C5—C6—C7	−179.13 (13)	C27—C28—C29—C30	168.80 (14)
O1—C6—C7—C8	−37.84 (18)	O2—C29—C30—C31	48.13 (18)
C5—C6—C7—C8	145.27 (12)	C28—C29—C30—C31	−131.89 (13)
O1—C6—C7—C16	84.85 (16)	O2—C29—C30—C39	−72.98 (17)
C5—C6—C7—C16	−92.03 (14)	C28—C29—C30—C39	107.00 (14)
C6—C7—C8—C9	−68.42 (15)	C29—C30—C31—C32	67.46 (15)
C16—C7—C8—C9	173.25 (11)	C39—C30—C31—C32	−172.94 (12)
C7—C8—C9—C10	86.49 (15)	C30—C31—C32—C33	−87.47 (16)
C7—C8—C9—C14	−92.62 (17)	C30—C31—C32—C37	94.23 (17)
C14—C9—C10—C11	0.9 (2)	C37—C32—C33—C34	0.2 (2)
C8—C9—C10—C11	−178.25 (13)	C31—C32—C33—C34	−178.18 (14)
C9—C10—C11—C12	−0.2 (2)	C32—C33—C34—C35	0.6 (2)
C10—C11—C12—C13	−0.7 (2)	C33—C34—C35—C36	−0.7 (2)
C11—C12—C13—C14	0.9 (2)	C34—C35—C36—C37	0.0 (2)
C12—C13—C14—C9	−0.2 (2)	C35—C36—C37—C32	0.8 (2)
C12—C13—C14—C15	−176.99 (14)	C35—C36—C37—C38	178.36 (14)
C10—C9—C14—C13	−0.7 (2)	C33—C32—C37—C36	−0.9 (2)
C8—C9—C14—C13	178.41 (13)	C31—C32—C37—C36	177.40 (13)
C10—C9—C14—C15	176.01 (14)	C33—C32—C37—C38	−178.41 (13)
C8—C9—C14—C15	−4.9 (2)	C31—C32—C37—C38	−0.1 (2)
C13—C14—C15—F3	−10.2 (2)	C36—C37—C38—F7	127.54 (15)
C9—C14—C15—F3	172.94 (13)	C32—C37—C38—F7	−54.91 (19)
C13—C14—C15—F1	−130.55 (15)	C36—C37—C38—F9	7.2 (2)
C9—C14—C15—F1	52.63 (19)	C32—C37—C38—F9	−175.27 (13)
C13—C14—C15—F2	109.57 (16)	C36—C37—C38—F8	−112.43 (15)
C9—C14—C15—F2	−67.26 (18)	C32—C37—C38—F8	65.13 (18)
C6—C7—C16—C17	164.93 (11)	C29—C30—C39—C40	−169.09 (11)
C8—C7—C16—C17	−72.64 (15)	C31—C30—C39—C40	70.02 (14)
C7—C16—C17—C22	115.21 (15)	C30—C39—C40—C41	68.56 (16)
C7—C16—C17—C18	−66.85 (17)	C30—C39—C40—C45	−109.77 (16)
C22—C17—C18—C19	−0.8 (2)	C45—C40—C41—C42	1.5 (2)
C16—C17—C18—C19	−178.86 (14)	C39—C40—C41—C42	−176.92 (14)
C17—C18—C19—C20	0.0 (3)	C40—C41—C42—C43	0.4 (2)
C18—C19—C20—C21	0.7 (3)	C41—C42—C43—C44	−1.7 (2)
C19—C20—C21—C22	−0.6 (3)	C42—C43—C44—C45	1.0 (2)
C20—C21—C22—C17	−0.2 (3)	C43—C44—C45—C40	1.0 (2)
C20—C21—C22—C23	177.92 (16)	C43—C44—C45—C46	−177.73 (14)
C18—C17—C22—C21	0.9 (2)	C41—C40—C45—C44	−2.2 (2)
C16—C17—C22—C21	178.83 (15)	C39—C40—C45—C44	176.09 (14)
C18—C17—C22—C23	−177.22 (13)	C41—C40—C45—C46	176.46 (13)
C16—C17—C22—C23	0.7 (2)	C39—C40—C45—C46	−5.2 (2)
C21—C22—C23—F6	−0.8 (2)	C44—C45—C46—F10	6.0 (2)
C17—C22—C23—F6	177.38 (14)	C40—C45—C46—F10	−172.73 (14)
C21—C22—C23—F4	−121.21 (16)	C44—C45—C46—F12	126.18 (16)
C17—C22—C23—F4	56.95 (19)	C40—C45—C46—F12	−52.6 (2)
C21—C22—C23—F5	119.02 (16)	C44—C45—C46—F11	−113.91 (16)
C17—C22—C23—F5	−62.8 (2)	C40—C45—C46—F11	67.36 (19)
